# Selection and Validation of Appropriate Reference Genes for qRT-PCR Analysis in *Isatis indigotica* Fort.

**DOI:** 10.3389/fpls.2017.01139

**Published:** 2017-06-28

**Authors:** Tao Li, Jing Wang, Miao Lu, Tianyi Zhang, Xinyun Qu, Zhezhi Wang

**Affiliations:** Key Laboratory of the Ministry of Education for Medicinal Resources and Natural Pharmaceutical Chemistry, National Engineering Laboratory for Resource Development of Endangered Crude Drugs in Northwest of China, Shannxi Normal UniversityXi’an, China

**Keywords:** qRT-PCR, reference gene, *Isatis indigotica* Fort., gene expression, normalization

## Abstract

Due to its sensitivity and specificity, real-time quantitative PCR (qRT-PCR) is a popular technique for investigating gene expression levels in plants. Based on the Minimum Information for Publication of Real-Time Quantitative PCR Experiments (MIQE) guidelines, it is necessary to select and validate putative appropriate reference genes for qRT-PCR normalization. In the current study, three algorithms, geNorm, NormFinder, and BestKeeper, were applied to assess the expression stability of 10 candidate reference genes across five different tissues and three different abiotic stresses in *Isatis indigotica* Fort. Additionally, the *IiYUC6* gene associated with IAA biosynthesis was applied to validate the candidate reference genes. The analysis results of the geNorm, NormFinder, and BestKeeper algorithms indicated certain differences for the different sample sets and different experiment conditions. Considering all of the algorithms, *PP2A-4* and *TUB4* were recommended as the most stable reference genes for total and different tissue samples, respectively. Moreover, *RPL15* and *PP2A-4* were considered to be the most suitable reference genes for abiotic stress treatments. The obtained experimental results might contribute to improved accuracy and credibility for the expression levels of target genes by qRT-PCR normalization in *I. indigotica.*

## Introduction

Gene expression analysis is an effective strategy that offers certain important information related to gene functions in the plant life cycle and the signal pathways that regulate effective component accumulations in response to environmental changes (Miao et al., 2015; Shu et al., 2015; Yue et al., 2015; Vojta et al., 2016). Selected methods can be applied to detect gene expression levels, including microarray/gene chips, RNA sequencing, semi-quantitative PCR (semi-qPCR), Northern blot, RNase protection analysis (RPA), and quantitative real-time PCR (qRT-PCR) (VanGuilder et al., 2008). Among these methods, qRT-PCR is considered one of the most popular techniques for analyzing gene expression levels due to its high sensitivity, specificity, reproducibility, accuracy, and high efficiency (Hu et al., 2014). However, qRT-PCR results are affected by the amount and source of the original sample, RNA integrity, cDNA quality and quantity, primer specificity, stability of the reference gene, efficiency of reverse transcription, and experimental conditions, among other factors (Kumar and Singh, 2015). To obtain reliable qRT-PCR analysis results, it is crucial to normalize the raw data for gene expression and minimize variations for the different samples and experimental conditions according to the Minimum Information for Publication of Real-Time Quantitative PCR Experiments (MIQE) guidelines (Han et al., 2012; Martins et al., 2016). These guidelines are most commonly used to select and normalize the reference gene for qRT-PCR analysis (Chao et al., 2012).

If qRT-PCR is used to analyze the target gene expression levels, the reference genes must be stably expressed for different samples and different experimental conditions (Wong and Medrano, 2005). In practice, the transcription levels of the traditional reference genes, even certain housekeeping genes, could not be expressed stably for different samples and experimental conditions (Kozera and Rapacz, 2013), i.e., no universal reference genes have been confirmed up to now (Radonić et al., 2004), a situation well documented in the published literature. For *Setaria viridis, Protein Kinase*, *RNA Binding Protein*, and *SDH* could be used as the most stable reference genes across different developmental stages and under drought or aluminum stress (Martins et al., 2016). For the tung tree, *ACT7*, *UBQ*, *GAPDH*, and *EF1*α were the most stable reference genes for all samples and developing seeds, and *ACT7*, *EF1*β, *GAPDH*, and *TEF1* were the top four candidates for different tissues, whereas *LCR69* and *ALB* did not favor qRT-PCR normalization under the same conditions (Han et al., 2012). For *Pinus massoniana* L., *ACT1* was confirmed as the optimal reference gene for all samples, *UBI4* was stably expressed in various tissues and under zinc stress, *CYP* was the best gene for insect-resistant leaves and Pb stress, and *Fbox* and *UBI11* could be used as the most stable reference genes for salt stress. Additionally, certain best reference groups have been identified, namely, *Fbox* + *RRP8*, *ARF* + *TUBA*, and *EF1B* + *IDH* for drought and low temperature stress and flowers in different developmental stages, respectively (Chen et al., 2016). Therefore, different reference genes should be used in different samples and experimental conditions. Furthermore, a reference gene with lower stability might lead to a biased understanding of qRT-PCR (Gutierrez et al., 2008) and mask the true nature of gene expression (Bustin, 2002; Bustin and Nolan, 2004).

Several prevalent mathematical algorithms are available, such as geNorm, NormFinder, and BestKeeper (Pfaffl, 2001; Vandesompele et al., 2002; Andersen et al., 2004), and can be used to explore novel reference genes for qRT-PCR. For geNorm and NormFinder analysis, all raw Ct values must be transferred to relative expression values according to the Delta Ct method, whereas no transformation is needed for BestKeeper. The geNorm algorithm considers both the average expression stability value (*M*-value) and pairwise variation (Vn/Vn+1) to assess the expression stability and determine the optimal number of the candidate reference genes (Vandesompele et al., 2002). NormFinder focuses attention on both intra- and inter-group variations and estimates the expression levels of the candidate reference genes using the stability value, with lower values indicating higher stability (Andersen et al., 2004). BestKeeper obtains the expression variability of reference genes through the standard deviation (SD), coefficient of variance (CV), or coefficient of correlation (Zhang W.X. et al., 2016). In general, different algorithms are usually combined together for qRT-PCR evaluation and normalization (Demidenko et al., 2011; Qi et al., 2016; Reddy et al., 2016).

The biennial herb *Isatis indigotica* Fort. (Cruciferae) is a traditional medical plant widely cultivated in China (Angelini et al., 2007) and is popular in pharmaceuticals, food additives, flavors, and other industrial materials due to abundant bio-active components, including alkaloids (Wu et al., 1997, 2011), flavonoids (Deng et al., 2008), organic acids, and glycosides (Liau et al., 2007). Until now, only a few references mentioned gene expression and functions in the developmental process and accumulation of metabolites responding to abiotic or biotic stress treatments in which the reference genes were nothing more than *UBQ*, *ACT* and 18s r RNA (Han et al., 2013; Li Q. et al., 2014; Chen et al., 2015; Dong et al., 2015; Han, 2015; Hu et al., 2015). The literature does not mention the selection and validation of accurate reference genes for gene expression in different samples and experimental conditions by qRT-PCR in this plant, nevertheless, it is a vital step for further gene function research.

In this study, 10 candidate reference genes (*UBC22, UBC29, Act7, PP2A-4, eIF2, APT3, AP-2, RPL15, TUB4*, and *UBC19*) from the transcriptome sequencing data in our lab were selected for validation of their expression stabilities for different tissues and abiotic stresses using qRT-PCR. Three mathematical algorithms (geNorm, NormFinder, and BestKeeper) were applied to analyze the raw Ct value and assess the reference gene expression stability. In addition, the candidate reference genes were tested using a target gene *IiYUC6*, which is involved in the pathway of indole-3-acetic acid (IAA) biosynthesis. According to the references (Yamamoto et al., 2007; Expósito-Rodríguez et al., 2011; Liu et al., 2014; Li et al., 2015), the expression patterns of *CsYUCs* in cucumber, *FvYUCs* in strawberry, *ToFZY* (YUCCA-like flavin monooxygenases, FMOs) in tomato, and *OsYUCCA* in rice were analyzed and the results revealed that the expression patterns of *YUCCA* varied in different species. Moreover, functional studies on *YUCCA6* were performed in strawberry (Liu et al., 2014), *Arabidopsis* (Kim et al., 2007; Im Kim et al., 2011; Cha et al., 2015), poplar (Ke et al., 2015), and potato (Im Kim et al., 2013). All of these research studies produced valuable references. Until recently, no research was available on *YUCCA* in *I. indigotica* Fort. or whether it played important roles in the synthesis of IAA, a topic that deserves further study. However, discussion of the expression patterns was the first step. In this work, we offer a full length CDS of *IiYUC6* in our transcriptome database that can be used to validate the candidate reference genes. The results are expected to act as a general guideline for reference gene selection in normalization and quantification of transcription levels for gene expression and function research in *I. indigotica*.

## Materials and Methods

### Plant Materials and Growth Conditions

Seeds of *I. indigotica* purchased from Shaanxi Geo-Authentic Medicinal Plant Co. Ltd. (Xi’an, China) were germinated on a seedling bed. When the first pair of true leaves appeared, the seedlings were transferred to pots filled with soil (4–5 seedlings per pot). The entire germination and growth process was conducted in the greenhouse in our lab under the conditions of a 16-18 h photoperiod, 2000 Lx irradiance level, 25 ± 2°C temperature, and 60–80% humidity. Furthermore, irrigation, regular weeding, and other management measures were performed every 2 days. Three-month-old seedlings were used as the abiotic treatment materials. At the same time, different tissues (including roots, stems, leaves, flowers, and fruits) were collected in the experimental field, frozen immediately in liquid nitrogen and stored at –80°C. All samples were gathered in three biological replications.

### Abiotic Stress Treatments

Three-month-old seedlings were randomly assigned to the control and treatment groups. Three types of abiotic stress treatments were applied: ABA, wounding, and cold stress treatments. The first treatment was performed using a foliar ABA spray solution (0.2 μM), and the control seedlings received water only. In addition, mechanical wounding treatment was applied by scratching approximately 40% of the total leaf surfaces with a blade (Ge et al., 2015). Finally, the cold treatments consisted of seedlings exposed to 4°C for different durations. For the wounding and cold treatment samples, the control groups received non-treatments. All of the above samples were harvested 6 h after the corresponding treatments, frozen into liquid nitrogen and stored at –80°C for further use.

### RNA Isolation and cDNA Synthesis

Total RNA was isolated from all samples using the TaKaRa MiniBEST Universal RNA Extraction Kit (TaKaRa, Dalian, China) with genomic DNA removed by the gDNA Eraser Spin Column and DNase I treatment according to the manufacturer’s instructions. The RNA integrity was examined using 1 % agarose gel electrophoresis. The concentration and purity of the total RNA were detected by a NanoDrop 2000c Spectrophotometer (Thermo Scientific, United States). The A_260_/A_280_ ratio of extracted total RNA ranged from 1.90 to 2.10, indicating high purity and no protein contamination. The first-strand cDNA was synthesized using the PrimeScript^TM^ II 1st Strand cDNA Synthesis Kit (TaKaRa) with 1.0 μg total RNA in a 20 μL volume according to the manufacturer’s protocols. Amplification using oligo (dT) 18 as primer was performed with the following thermal cycling: 42°C for 50 min and 70°C for 10 min. The RT-PCR products were diluted (1:20) for qRT-PCR assays.

### Selection of Candidate Reference Genes and Primer Design

Based on previous research studies and the transcriptome sequencing data of our lab (unpublished), 10 candidate genes were selected to normalize and validate the qRT-PCR, including 3 homologous genes (*UBC19, UBC22*, and *UBC29*), 2 traditional housekeeping genes (*ACT7* and *TUB4*) and five novel genes (*PP2A-4, eIF2, APT3, AP-2, RPL15*). To ensure the reliability and accuracy of the putative reference genes, the nucleotide sequences of 10 candidate genes were BLAST-searched against the *Arabidopsis* genome database to identify their homologs in *I. indigotica.* Forward and reverse primers of all candidate genes were designed using *Primer Premier v5.0* software using Tm values ranging from 58°C to 62°C, GC % (45–60), primer length (19–23 bp), and product length (85–225 bp). All primers were synthesized by Beijing Genomics Institute. The ortholog sequences of *Arabidopsis*, primer information, and gene characteristics are presented in **Table 1**. Moreover, the primer specificity was validated using melting curve analysis.

**Table 1:** Details of the primers used for qRT-PCR normalization.

Gene abbreviations	Gene name	Accession No.	Length (bp)	Primer sequence U/L [5′–3′]	*Arabidopsis* ortholog No	Identity (%)	Tm°C	*E* (%)	R^2^
*eIF2*	Eukaryotic transation initiation factor	c40298.graph_c0	117	For:TACCAGTGGCTCGCTTGAC	At5g20920.3	87	83	1.20	0.9583
				Rev:CAACCAAAGCAAATGACGTACTC					
*ACT7*	Actin7	AY870652.1	181	For: AGGAATCGCTGACCGTATG	At5g09810.1	93	85.5	1.15	0.9829
				Rev : TGGACCCGACTCATCGTATT					
*PP2A-4*	Protein phosphatase 2A-4	c41201.graph_c0	151	For: GAATGCCTGCGAAAGTATGG	At3g58500.1	93	83	1.12	0.9859
				Rev: TCCTAATGTTGTCAAGGGTCTC					
*AP-2*	AP-2 complex	c47959.graph_c0	137	For: AAGACCTTCAGCCTTACGCC	At5g22780.1	92	83	1.35	0.9849
				Rev: ACTGCATCGAGGTTGTCTGG					
*APT3*	Adenine phosphoribosyl transferase	c37104.graph_c0	178	For:CCGTGTCGTTCCAGATTTTC	At4g22570.1	93	82	1.04	0.9983
				Rev:GATTGGTGGACCGAATAGGA					
*UBC29*	Ubiquitin-conjugating enzyme 29	c38774.graph_c0	148	For:TTCTTAAAGACCAGTGGAGC	At2g16740.1	90	83.5	0.97	0.9996
				Rev:ATGGCTTCGTATTTGACCTT					
*RPL15*	Ribosomal protein L	c42633.graph_c1	133	For:GGGCGAAGAGGAAAGGTAG	At3g25920.1	87	84.6	1.05	0.9920
				Rev: CGGAAGACGGCGATAAAGAG					
*UBC22*	Ubiquitin-conjugating enzyme 22	c38263.graph_c0	225	For:GTATGAAGTTGGCATTGTCGC	At5g05080.2	89	83	0.89	0.9990
				Rev:TCTTTCCAGCCTGTTCGTTT					
*UBC19*	Ubiquitin-conjugating enzyme 19	c39354.graph_c0	188	For: TGAGACATGCTGCTTCCATC	At3g20060.1	92	84	0.94	0.9845
				Rev: CTTCTTGGTTGCTCCAAAGC					
*TUB4*	Tubulin	c43494.graph_c0	85	For:GCCCCGATAACTTCGTCTT	At5g44340.1	93	85.8	1.12	0.9790
				Rev: TCAATCAACTCCGCTCCCT					


### qRT-PCR Conditions and Amplification Efficiency

The qRT-PCR was performed on a 7500 ABI Real-time PCR system (Applied Biosystems, United States) using SYBR Premix Ex Taq (Takara Bio, Kusatsu, Shiga, Japan). The total 20 μL reaction mixture, including 10 μL SYBR Premix Ex Taq, 2 μL cDNA, 0.8 μL forward primer, 0.8 μL reverse primer, and 6.4 μL dd H_2_O in a 96-well optical plate, was amplified according to the following thermal cycling conditions: 95°C for 10 s, followed by 40 cycles of 5 s at 95°C and 30 s at 60°C. To draw the melting curve, the PCR product was heated from 65 to 95°C (0.5°C/5 s), and the raw Ct values were obtained. Each qRT-PCR reaction was conducted in technical triplicate. The slope of the standard curve generated through serial 10-fold dilutions of the cDNA samples was used to calculate the amplification efficiency for each candidate gene.

### Assessment of the Expression Stability of the Reference Gene

Three software packages, namely, geNorm, NormFinder, and BestKeeper, were used to analyze the gene expression stability based on their own algorithms. geNorm assesses the expression stability of the candidate reference gene using the *M*-value, which refers to the average pairwise variation between each reference gene and the other reference genes. A gene with *M* < 1.5 is usually treated as the stable reference gene (Guo et al., 2010; Han et al., 2012). Furthermore, the normalization factor obtained by calculating the pairwise variation (Vn/Vn+1) of the two sequential normalization factors (NFn and NFn+1) can be used to determine the most suitable numbers of the reference genes. If the value of Vn/Vn+1 is lower than 0.15, then the value of “*n*” is the most appropriate number of reference genes. The stability value is the important factor for NormFinder in estimating the top internal control genes, and the intra- and inter-group variations are also necessary factors that must be considered. The lower the stability value and inter- and intra-group variation, the more stable the reference gene will be. The SD value set at 1.0 by BestKeeper represents the Ct variation. If the value is less than 1.0, the candidate gene is considered a stable inter-control gene and vice versa. Furthermore, the coefficient of variation (CV value) is used to rank the candidate reference genes.

### Validation of the Candidate Reference Genes

The selected reference genes, including the most and least stable genes, were validated for different tissue samples (root, stem, leaf, flower and fruit) using the relative expression level analysis of *IiYUC6*, a key gene associated with IAA biosynthesis in *I. indigotia*. The 2^-ΔΔct^ method was used to calculated the relative expression level of the target gene.

### Statistical Analysis

Statistical analyses were performed using SPSS 22.0, and all data for the relative expression level of the target gene were subjected to one-way analysis of variance (ANOVA) and presented as the means ± standard error (SE). The statistical level was assessed using ^∗^*P* < 0.05, ^∗∗^*P* < 0.01, and ^∗∗∗^*P* < 0.001.

## Results

### Primer Specificity and Expression Level Analysis of 10 Candidate Reference Genes

**FIGURE 1:**
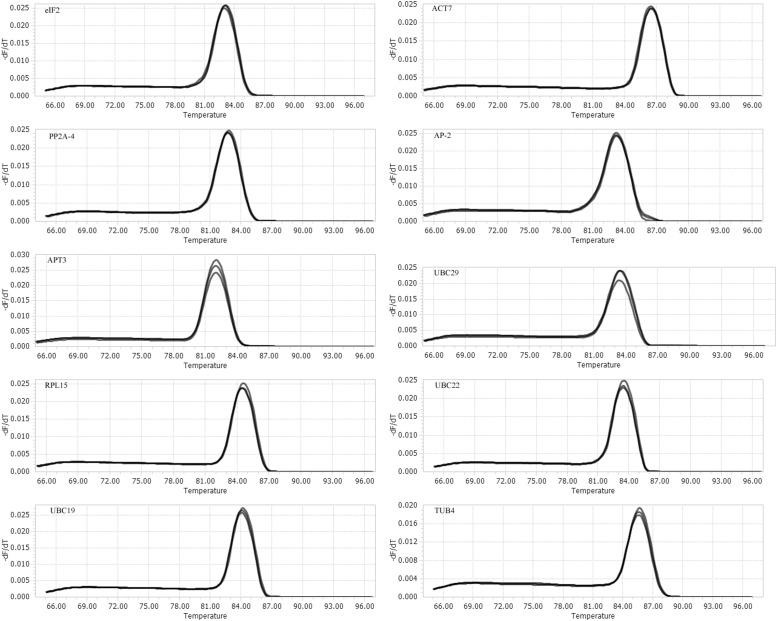
Melting curves for the candidate reference genes. Melting temperatures were visualized by plotting the negative first derivative of fluorescence relative to the temperature in Celsius (–(d/dT)). Melting curves of 10 reference genes show a simple peak.

**FIGURE 2:**
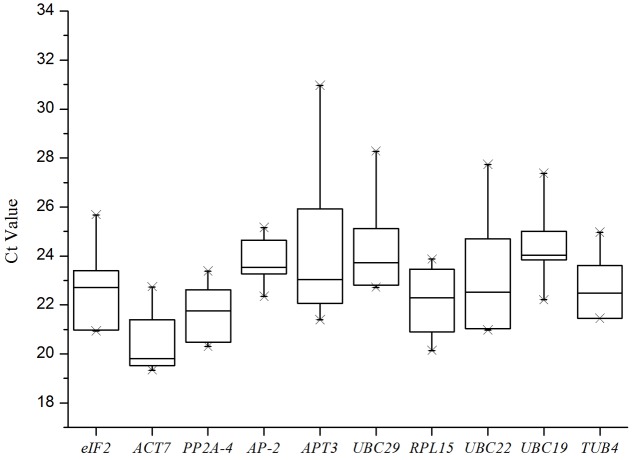
Ct values for 10 candidate reference genes. Expression data are displayed as Ct values for each reference gene in all combined experimental conditions. A line across the box depicts the median. The vertical sides of the box indicate the 25th and 75th percentiles, and the whiskers show the caps for maximum and minimum values.

A total of 10 putative reference genes (*UBC19, UBC22, UBC29, ACT7, PP2A-4, eIF2, APT3, AP-2, RPL15*, and *TUB4*) from the transcriptome sequencing data of *I. indigotica* were selected as candidates for qRT-PCR normalization. The gene names and abbreviation, accession number, primer sequences, *Arabidopsis* orthologous, identity (compared to *Arabidopsis*), amplification efficiency (E) and length, Tm value and correlation coefficient (*R*^2^) are listed in **Table 1**. The Tm values ranged from 83°C to 85.8°C, the amplification efficiency varied from 89% for *UBC22* to 135% for *AP-2*, and the correlation coefficients (*R*^2^) ranged from 0.9583 for *eIF2* to 0.9996 for *UBC29*. Moreover, the amplifications were validated using the single peak of the melting curve for each primer (**Figure 1**). The gene expression levels were determined by the cycle threshold (Ct) values, and the Ct value distribution of the 10 candidate reference genes varied from 19.34 to 30.97 (**Figure 2**), which represents the different expression levels of the candidates. Among them, *APT3* (for wounding stress treatment) showed the lowest expression level with the highest Ct value (30.97), and *ACT7* (in root) presented the highest expression level with the lowest Ct value (19.34).

### Expression Stability Analysis of 10 Candidate Reference Genes

The performance of 10 candidate reference genes in *I. indigotica* was assessed via qRT-PCR in different samples, which were divided into three experimental sets (total, tissues and stresses), five different tissues (roots, stems, leaves, flowers, and fruits), and three stress treatments (ABA, wounding, and cold).

**FIGURE 3:**
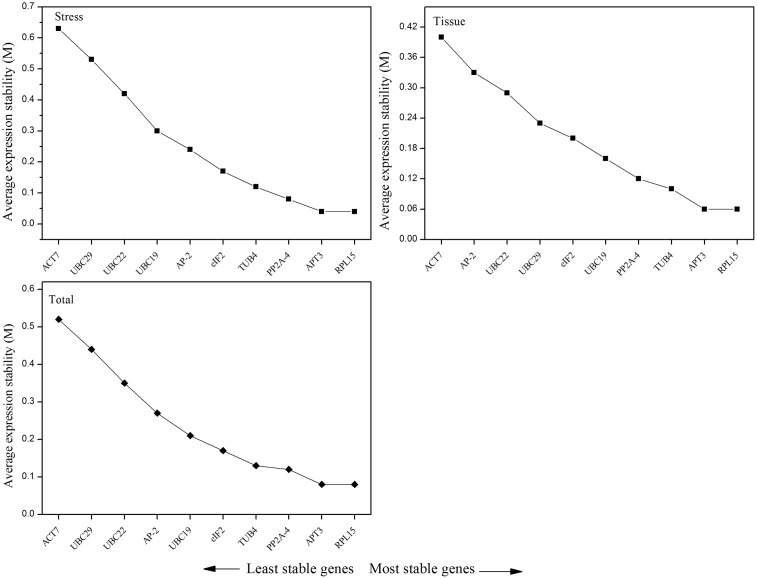
Average expression stability (*M*-value) of 10 candidate reference genes calculated by geNorm and ranking of the candidate reference genes in different tissues, abiotic treatments, and total group, respectively. A lower value of average expression stability (*M*) indicates a more stable expression level.

According to the *M*-value, geNorm depicted the ranking of 10 candidate reference genes for the different tissues and experimental conditions (**Table 2** and **Figure 3**). For the five tissue samples, the expression stability ranking of the 10 reference genes was arranged as follows: *RPL15 > APT3 > TUB4 > PP2A-4 > UBC19 > eIF2 > UBC29 > UBC22 > AP-2 > ACT7.* For the abiotic stress samples, the most stable and relatively unstable genes were *RPL15* (*M* = 0.04) and *ACT7* (*M* = 0.63), respectively. Based on all data sets, *RPL15* (*M* = 0.08) was still the most stable reference gene, followed by *APT3* (M = 0.08) and *PP2A-4* (*M* = 0.12). Additionally, *ACT7* (*M* = 0.52) was the most variable reference gene for the same samples and experimental conditions (**Table 2**).

**Table 2:** Ranking of the candidate reference genes and their *M*-values calculated by geNorm.

	Total		Tissue		Abiotic stress	
			
Rank	Gene	*M*-value	Gene	*M*-value	Gene	*M*-value
1	*RPL15*	0.08	*RPL15*	0.06	*RPL15*	0.04
2	*APT3*	0.08	*APT3*	0.06	*APT3*	0.04
3	*PP2A-4*	0.12	*TUB4*	0.10	*PP2A-4*	0.08
4	*TUB4*	0.13	*PP2A-4*	0.12	*TUB4*	0.12
5	*eIF2*	0.17	*UBC19*	0.16	*eIF2*	0.17
6	*UBC19*	0.21	*eIF2*	0.20	*AP-2*	0.24
7	*AP-2*	0.27	*UBC29*	0.23	*UBC19*	0.30
8	*UBC22*	0.35	*UBC22*	0.29	*UBC22*	0.42
9	*UBC29*	0.44	*AP-2*	0.33	*UBC29*	0.53
10	*ACT7*	0.52	*ACT7*	0.40	*ACT7*	0.63


**Table 3** shows the ranking of the 10 candidate reference genes and their expression stability values as calculated by NormFinder. *RPL15* and *APT3* were the top two most suitable genes for normalization if both different tissue (stability value = 0.019) and abiotic stress (stability value = 0.011) samples were considered. Moreover, according to all data sets, *RPL15* (stability value = 0.038) and *PP2A-4* (stability value = 0.073) were the top two best reference genes for normalization of qRT-PCR, but the most variable reference gene was *UBC29* (stability value = 0.571).

**Table 3:** Ranking of the candidate reference genes and their expression stability values calculated by NormFinder.

	Total		Tissue		Abiotic stress	
			
Rank	Gene	Stability	Gene	Stability	Gene	Stability
1	*RPL15*	0.038	*RPL15*	0.019	*RPL15*	0.011
2	*PP2A-4*	0.073	*APT3*	0.019	*APT3*	0.011
3	*APT3*	0.076	*TUB4*	0.023	*PP2A-4*	0.023
4	*eIF2*	0.097	*PP2A-4*	0.042	*TUB4*	0.030
5	*TUB4*	0.105	*eIF2*	0.125	*eIF2*	0.046
6	*AP-2*	0.149	*UBC29*	0.142	*UBC19*	0.189
7	*UBC19*	0.154	*UBC19*	0.158	*AP-2*	0.224
8	*ACT7*	0.250	*AP-2*	0.189	*ACT7*	0.389
9	*UBC22*	0.337	*ACT7*	0.269	*UBC22*	0.761
10	*UBC29*	0.571	*UBC22*	0.392	*UBC29*	1.151


In addition, the ranking results examined by BestKeeper are presented in **Table 4** based on calculation of the CV and SD values. The results revealed that *AP-2* (CV = 3.08, *SD* = 0.75) and *PP2A-4* (CV = 4.30, *SD* = 0.94) were the first and second most stable reference genes for the abiotic stress samples. *UBC29* (CV = 2.02, *SD* = 0.47) was the most stable reference gene, followed by *AP-2* (CV = 3.06, *SD* = 0.72) if different tissue samples were considered. According to all data sets, the top two most stable reference genes were *AP-2* (CV = 3.49, *SD* = 0.84) and *PP2A-4* (CV = 4.47, *SD* = 0.97), and the least stable reference gene was *APT3* (CV = 10.41, *SD* = 2.59).

**Table 4:** Ranking of the candidate reference genes and their expression stability values calculated using BestKeeper.

Gene		UBC29	AP-2	TUB4	ACT7	UBC19	APT3	eIF2	PP2A-4	RPL15	UBC22
Total	SD[±CP]	1.46	**0.84**	1.10	1.07	1.10	2.59	1.14	**0.97**	1.08	1.94
	CV	5.96	3.49	4.80	5.18	4.47	10.41	5.01	4.47	4.82	8.32
Tissue	SD[±CP]	**0.47**	0.72	**0.76**	**0.81**	**0.86**	**0.86**	**0.99**	**0.99**	1.09	1.63
	CV	2.02	3.06	3.37	3.99	3.59	3.77	4.47	4.57	4.85	7.11
Abiotic stress	SD[±CP]	1.20	**0.75**	1.38	1.10	1.31	1.76	1.16	**0.94**	1.01	2.54
	CV	4.54	3.08	5.86	5.14	5.15	6.22	4.86	4.30	4.51	10.62


*The value of SD[±CP] below 1.0, marked in bold, indicate that the reference gene is qualified, and the value of CV is used to rank the reference genes.*

**FIGURE 4:**
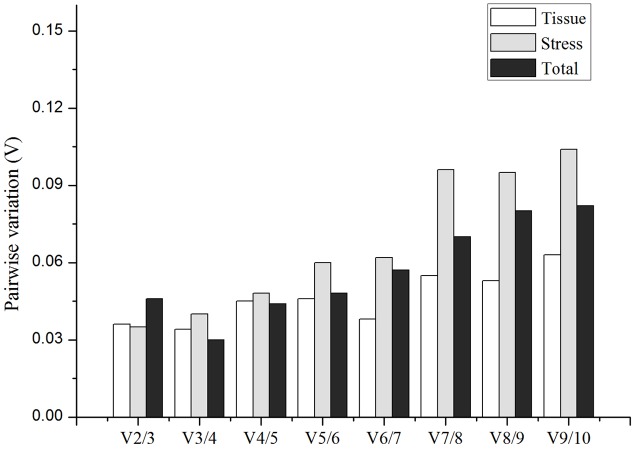
Pairwise variation (*V*-value) calculated by geNorm showing the optimal number of reference genes required for qRT-PCR normalization in different tissues, abiotic treatments and total group, respectively. The average pairwise variations Vn/Vn+1 were analyzed between the normalization factors NFn and NFn+1.

Furthermore, after identification of the best candidate gene for each experimental set, the pairwise variations Vn/Vn+1 were investigated by geNorm to normalize qRT-PCR for the different tissues and abiotic stresses (**Figure 4**). The results revealed that the V2/3 value between the two sequential normalization factors (NF2 and NF3) was the lowest and was less than the cutoff threshold of 0.15 in all of the experimental sets. Therefore, the optimal number of the candidate reference genes should be 2, showing that the best combination of the reference genes for normalization of qRT-PCR were *RPL15* and *TUB4* for different tissues and *RPL15* and *PP2A-4* for abiotic stress samples in *I. indigotica.*

### Validation of the Selected Reference Genes for Different Tissues

To evaluate the effects of suitable candidate reference genes on assessment of target gene expression, we examined the expression patterns of *I. indigotica YUCCA* (*IiYUC6*), a key gene involved in IAA synthesis (Kim et al., 2007) in different tissues. The relative expression levels of the three top ranking reference genes (*RRL15*, *APT3*, and *TUB4*) and two least stable genes (*ACT7* and *UBC22*) were validated according to geNorm and NormFinder.

**FIGURE 5:**
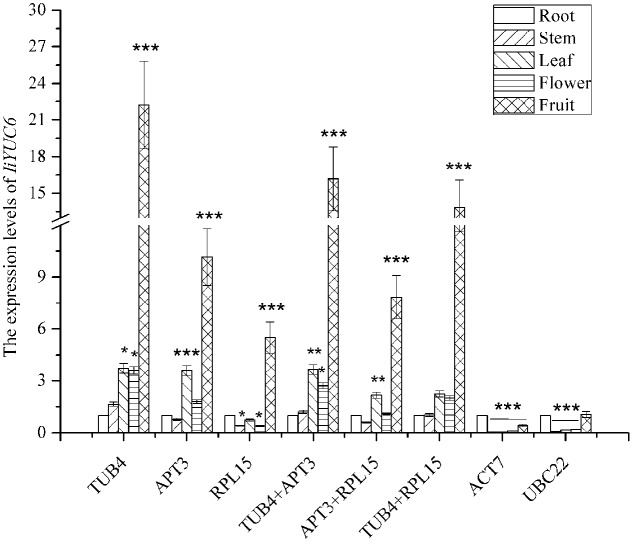
Relative expression levels of *IiYUC6* in different tissues (root, stem, leaf, flower, and fruit) were normalized using different reference genes or a combination including the three most stable (*TUB4*, *APT3*, and *RPL15*) and two least stable genes (*ACT7* and *UBC22*). Error bars show the mean standard error calculated from three biological replicates. The statistical level was assessed with ^∗^*P* < 0.05, ^∗∗^*P* < 0.01, and ^∗∗∗^*P* < 0.001.

As shown in **Figure 5**, the transcription level of *IiYUC6* was upregulated compared with the results in root when normalized with *TUB4.* Similar expression patterns were observed when normalized by *TUB4* combined with *APT3*. When normalized with *APT3*, the expression level of *IiYUC6* was still upregulated in most of the tissues except for stem, but the difference was not apparent. However, downregulation of the transcription level of *IiYUC6* was noted in stem, leaf and root when normalized with *RPL15*, and similar expression patterns were also observed when normalized with *APT3* and *RPL15.* In addition, the highest expression level occurred for *IiYUC6* in fruit when normalized with the most stable reference genes, including *TUB4, APT3*, and *RPL15* or their pairwise combinations. In contrast, when normalized with the least stable reference genes of *ACT7* or *UBC22*, the expression profiles were more variable, with obvious downregulated expression levels in different tissues compared with the root sample. In conclusion, similar expression levels of the target gene were obtained when normalized with *RPL15*, *APT3*, and *TUB4*. However, when unstable reference genes including *ACT7* and *UBC22* were used, the relative expression level was underestimated, which might lead to a biased result. Hence, the analysis results of expression patterns on *IiYUC6* revealed that the candidate reference gene selection was feasible and credible.

## Discussion

Currently, qRT-PCR plays an important role in quantifying gene expression level because of its sensitivity, specificity, reproducibility and accuracy (Bustin, 2002; Bustin et al., 2005; Nolan et al., 2006). To obtain reliable results, the appropriate reference gene must be selected, which is a key factor in assessing the expression level of the target gene by qRT-PCR. In previous studies, certain traditional reference genes were selected and validated for different samples and various experimental conditions in certain species (Kumar et al., 2011), such as in *Arabidopsis thaliana* (Czechowski et al., 2005; Dekkers et al., 2012), *Salvia miltiorrhiza* (Yang et al., 2010), *Glycine max* (Jian et al., 2008; Libault et al., 2008), *Brachiaria grass* (Silveira et al., 2009), *Cucumis sativus* (Wan et al., 2010), etc. Until now, no systematic study was available on the reliable reference gene selection and validation for qRT-PCR normalization in *I. indigotica*. Previously, *18S rRNA* (Hu et al., 2015), *IiActin* (Han et al., 2013; Li Q. et al., 2014; Dong et al., 2015; Han, 2015) and *Ubiquitin* (Chen et al., 2015) had been used as internal control genes to normalize the target gene (*IiFeSOD, IiHCT, IiGGPPS1*, and selected relative genes involved in biosynthesis of lignans) expression levels in *I. indigotica.* However, it is well known that a universal reference gene does not exist. Therefore, it is important and necessary to select and validate the best suitable reference genes for target gene function research in *I. indigotica.*

RNA-seq is one of the most effective high-throughput sequencing methods, and the sequencing data can be used to explore the specific functional genes or candidate reference genes. For instance, suitable reference genes were selected and validated from the transcriptome data of *Gentiana macrophylla* (He et al., 2016), *Fagopyrum esculentum* (Demidenko et al., 2011), and *Pinus massoniana* (Chen et al., 2016). Therefore, the reported literature on the transcriptome information of *I. indigotica* (Chen et al., 2013; Tang et al., 2014; Chen et al., 2015; Zhou et al., 2015; Zhang L. et al., 2016), combined with our lab’s transcriptome data on this plant (unpublished), had well served as the effective resources for finding the candidate reference genes. In this work, 10 candidate reference genes were chosen, and their expression stabilities were validated for five different tissues and three abiotic stress treatments for qRT-PCR normalization.

In the current paper, the candidate reference genes primarily included 3 homologous genes (*UBC19, UBC22*, and *UBC29*), 2 traditional housekeeping genes (*ACT7* and *TUB4*) and 5 other novel genes (*PP2A-4, eIF2, APT3, AP-2, RPL15*). Among these, three homologous genes, i.e., *UBC19, UBC22*, and *UBC29*, played different roles in normalization of qRT-PCR, and *UBC22* and *UBC29* were used as the least stable reference genes, but *UBC19* was the moderate performer. Furthermore, as novel reference genes, *RPL15* and *PP2A-4* were the most stable candidate reference genes for different tissues and experimental conditions. As traditional housekeeping genes, *ACT7* and *TUB4* were average performers and the same as the other novel genes, such as *eIF2* and *AP-2. APT3*, which was an ambivalent gene, ranked in the second and third places for geNorm and NormFinder, respectively, whereas it showed the least performance for BestKeeper. Therefore, the different candidate reference genes showed different expression characteristics for the different tissues and experimental conditions according to the corresponding algorithms in *I. indigotica*.

Homologous genes have been commonly used as reference genes for gene expression analysis. Taking the *Actin* gene in *Fortunella crassifolia* as an example, *ACT6*, *ACT8*, and *ACT7* were selected as internal control genes for salt, drought and heavy metal stress treatments, respectively (Hu et al., 2014). For *Glycine max*, the expressions of *EF1A 2a*, *EF1A 2b*, and *EF1A1a1* were the most stable under all tested conditions and therefore, these genes were always included in all gene combinations (Costa et al., 2016). However, our experimental results revealed that *UBC19, UBC22*, and *UBC29*, as homologous genes, exhibited different expression levels and relatively least stabilities, especially *UBC22* and *UBC29.* Hence, although the homologous genes have similar sequences, they play various roles in gene expression quantification.

*PP2A-4* heterotrimeric protein phosphatase is a ubiquitous and conserved serine/threonine phosphatase with broad substrate specificity and diverse cellular functions. As another suitable reference gene in the current paper, *PP2A-4* was a good performer for different tissues and abiotic stress treatments in *I. indigotica*. Moreover, *LsPP2A-1* was the most stable in diurnal and developmental timecourse experiments in *Lactuca sativa* (Sgamma et al., 2016). Similar results were observed in *Malus domestica, PP2A-4* was recommended as the most suitable reference gene for all tissues and different biotic stresses (Kumar and Singh, 2015) and for abiotic stress and all sample sets in *Sorghum bicolor* (Reddy et al., 2016). Additionally, *PP2Acs* was the best performer in heat and waterlogging stress in Chrysanthemum (Gu et al., 2011). In general, *PP2A-4* could be a potential reference gene for optimization of qRT-PCR normalization.

In conjunction with rRNA, ribosomal protein makes up the ribosomal subunits involved in the cellular process of translation, and its gene is ubiquitously expressed and often used as a good reference gene. *RPL2* was found to be the best reference gene for all tissue types and different biotic treatments in *Malus domestica* (Kumar and Singh, 2015). In the same way, *RPL15* exhibited the best stable expression levels for different tissues and abiotic stress treatments in *I. indigotica*. However, *RPL* was considered the most unstable gene across the developmental stages of *P. tomentosa* stems (Wang et al., 2016). In summary, the ribosomal protein showed expression diversity for different species and different experimental conditions, and our findings enrich the overall knowledge of ribosomal protein expression patterns.

*ACT7*, a member of the *Actin* gene families (Ge et al., 2015; Tian et al., 2015), is commonly used as a common reference gene but showed the least stability for different tissues and abiotic stresses according to geNorm in this study. *TUB4*, a member of the *Tubulin* gene families, is also used as common reference gene for qRT-PCR analysis, but for *I. indigotica*, it presented different expression characteristics for different tissues and abiotic stresses. The results revealed that not all of the common reference genes can be used as suitable internal control genes without selection and validation, and the same reference gene might have different expression levels for different samples and experiment conditions.

The three algorithms of geNorm, NormFinder and BestKeeper were applied to identify the suitable candidate reference genes in this study. Previous research reported the ranking results of suitable reference genes according to different software analyses (Wang et al., 2016), and the candidates of reference genes selected by different algorithms were varied (Li et al., 2016). Our results revealed that the top three rankings for the candidate reference gene were *RPL15*, *APT3*, and *PP2A-4* for all samples according to geNorm and were *RPL15*, *PP2A-4*, and *APT3* according to NormFinder. Nevertheless, *PP2A-4* remained in second place, and first place went to *AP-2* in BestKeeper analysis. The ranking order calculated by BestKeeper was slightly different from the results generated by geNorm and NormFinder, and a similar result was found by Qi’s research (Qi et al., 2016). *PP2A-4* was considered the acceptable reference gene for all samples according to the three algorithms. However, it is worth mentioning that the stability ranking of *PP2A-4* was slightly inconsistent if considering different tissues and different abiotic stress samples.

*YUCCA* (*YUC*) encodes FMOs that could convert indole-3-pyruvate (IPA) to indole-3-acetic acid (IAA) and was identified in *Arabidopsis* (Kim et al., 2007), *Zea mays* (Li et al., 2015), *Cucumis sativus* (Yan et al., 2016), *Glycine max* (Costa et al., 2016), *Triticum aestivum* (Li N. et al., 2014), Fragaria × ananassa Duch (Liu et al., 2012), and *Oryza sativa* (Abu-Zaitoon, 2014). Based on YUCCA protein, a similar sequence from the transcriptome data of *I. indigotia* was found in the Full-Length CDS DataBase, and the expression patterns of *IiYUC6* were discussed. In this work, *IiYUC6* presented the highest expression level in fruit compared with other tissues. Similar results were observed from strawberry in which *FaYUC1-2* might be involved in flower and fruit development processes (Liu et al., 2012). Therefore, our results suggested that *IiYUC6* could play an important role in fruit development processes in *I. indigotica*.

In brief, this work examined the selection and validation of candidate reference genes for qRT-PCR normalization for different tissues and abiotic stress treatments in *I. indigotica*. Identification of a suitable reference gene depends on the types of tissues, experimental conditions and analysis methods, and therefore, it is vital and essential to highlight the appropriate reference genes for corresponding samples in different species. The obtained results offer effective information related to the excavation and functional analysis of important gene resources in *I. indigotica*.

## Author Contributions

TL managed and organized the experimental studies. JW performed the qRT-PCR experiments and led the overall data analysis. ML designed the primers and performed the qRT-PCR. TZ and XQ directed the abiotic stress treatments. TL and JW drafted the manuscript, and ZW revised it. All authors discussed and commented on the manuscript.

## Conflict of Interest Statement

The authors declare that the research was conducted in the absence of any commercial or financial relationships that could be construed as a potential conflict of interest.
